# More T cell receptors to the RAScue in cancer?

**DOI:** 10.1172/JCI184782

**Published:** 2024-11-01

**Authors:** Eric Tran

**Affiliations:** Earle A. Chiles Research Institute, a division of Providence Cancer Institute, Portland, Oregon, USA.

## Abstract

Treatment with T cells genetically engineered to express tumor-reactive T cell receptors (TCRs), known as TCR-gene therapy (TCR-T), is a promising immunotherapeutic approach for patients with cancer. The identification of optimal TCRs to use and tumor antigens to target are key considerations for TCR-T. In this issue of the *JCI*, Bear and colleagues report on their use of in vitro assays to characterize four HLA-A*03:01– or HLA-A*11:01–restricted TCRs targeting the oncogenic KRAS G12V mutation. The TCRs were derived from healthy donors or patients with pancreatic cancer who had received a vaccine against mutant KRAS. The most promising TCRs warrant testing in patients with KRAS G12V–positive cancers.

## T cell receptor–based cell therapies against solid cancers

The administration of in vitro–expanded tumor-infiltrating lymphocytes (TILs) was the first T cell receptor–based (TCR-based) cellular therapy to target solid cancers. This approach was pioneered by Rosenberg and colleagues at the National Cancer Institute (NCI) (1) and entails obtaining tumor material from a patient, followed by large-scale in vitro expansion of T cells within the TIL and infusion of these cells into the patient followed by interleukin-2 (2). Notably, this therapy can mediate durable clinical responses in some patients with metastatic melanoma (3–5), which led to its approval by the FDA for this indication in February 2024. TIL therapy also has mediated clinical responses in epithelial-derived cancers, and like in melanoma, responses largely were driven by T cells harboring TCRs that target tumor antigens, such as neoantigens derived from cancer mutations (6).

The ability to isolate tumor-reactive TCR sequences and advances in T cell engineering permitted the development of TCR-gene therapy (TCR-T), a strategy that involves the in vitro genetic insertion of a tumor antigen–reactive TCR into the peripheral blood T cells of a patient, followed by expansion and infusion of the gene-modified T cells back into the patient (2). This process can generate a large number of tumor-antigen–reactive T cells. Unlike TIL therapy, which can only be used to treat the autologous patient, TCR-T allows for an “off-the-shelf” TCR that can be used to treat any patient whose tumors express the targeted antigen and matching HLA-restriction element of the TCR.

The targeted antigen is a key consideration for TCR-T; optimal tumor antigens are tumor specific, to avoid toxicity in healthy tissue, and essential for maintenance of the malignant phenotype. Oncogenic viruses and driver mutations fulfill these criteria. Indeed, regression of metastatic epithelial-derived cancer has been observed in patients receiving TCR-T targeting oncogenic HPV16-E6 or E7 (7, 8) or the hotspot mutations KRAS G12D (9) or TP53 R175H (10). A major limitation of TCR-T, however, is that a given TCR only can treat an often-small subset of patients due to the dual requirement of the specific tumor antigen and HLA expression. For example, less than 5% of USA White and Black patients with pancreatic ductal adenocarcinoma (PDAC) would be eligible for TCR-T using the HLA-C*08:02–restricted KRAS G12D targeting TCRs of Leidner and colleagues (9). One strategy to extend TCR-T eligibility to more patients is to identify additional TCRs that target tumor antigens restricted by different HLA, thereby building a library of off-the-shelf TCRs.

## Identification and characterization of mutant KRAS-reactive TCRs

In this issue of the *JCI*, Bear et al. (11) add to the expanding library of promising TCRs targeting oncogenic KRAS mutations (12–15). The authors further characterize two TCRs from their past study (16) derived from healthy donors’ peripheral blood T cells. These TCRs targeted KRAS G12V in the context of HLA-A*03:01 or HLA-A*11:01, which are common HLA alleles found in individuals identified as White, Black, and Asian in the US. Bear and authors also reported an additional KRAS G12V-reactive, HLA-A*11:01–restricted TCR from one of the healthy donors. Notably, the authors explored the potential of isolating mutant KRAS–reactive (mKRAS-reactive) TCRs from patients who received a mKRAS vaccine in a phase 1 trial. The vaccine comprised an autologous mature DC vaccine given intravenously in the adjuvant setting in patients with PDAC. Nine patients received two doses of the vaccine (prime and boost), which consisted of DCs exposed to (aka pulsed with) various long and/or short mKRAS peptides. Some patients received a vaccine that contained other KRAS G12 mutations in addition to the KRAS G12 mutation expressed by the autologous tumor. The vaccine was safe, with no grade 3 adverse events observed, and five of nine patients were alive at the median follow-up of about 25 months. The vaccine elicited T cell responses against mKRAS in six of nine patients as determined by IFN-γ ELISPOT assay, and the inclusion of long mKRAS peptides appeared to enhance immunogenicity of the vaccine, likely through stimulation of CD4^+^ T cells.

In an exemplary case, a patient with the HLA-A*03:01 and HLA-A*11:01 genotype was vaccinated with mature DCs pulsed with a nonamer (nine amino acid) and decamer (10 amino acid) KRAS G12V peptide. Two weeks later, the authors detected an elevated frequency of T cells in the peripheral blood that specifically recognized mKRAS G12V and not wild-type KRAS in the context of HLA-A*11:01. A mKRAS-reactive TCR sequence was isolated from an oligoclonal population of T cells in this patient (designated A11Vc).

This TCR alongside three other KRAS G12V–reactive TCRs derived from peripheral blood T cells of two healthy donors underwent in vitro testing for specificity, cross-reactivity, functional avidity, function in CD4^+^ T cells (CD8 coreceptor dependency), and tumor cell-line recognition. Three of the four KRAS G12V-reactive TCRs were restricted by HLA-A*11:01 (A11Va, A11Vb, and A11Vc), while one TCR was restricted by HLA-A*03:01 (A3V). All four TCRs did not recognize wild-type KRAS, but interestingly, the three HLA-A*11:01–restricted TCRs recognized the KRAS G12C peptide, albeit at 10-to-100-fold lower potency compared with the KRAS G12V peptide. The authors did not identify any concerning cross-reactivity of the TCRs against a select panel of peptides derived from the human proteome that were structurally related to the G12V peptide.

Coculture experiments of the TCR-engineered T cells with peptide-pulsed antigen-presenting cells and various tumor cell lines as targets revealed that T cells with TCR A11Va had high functional avidity and were the most potent at killing tumor cell lines in vitro. In CD4^+^ T cells, the three HLA-A*11:01 restricted TCRs were partially functional while the A3V TCR was not functional, which has implications if the TCR is to be introduced into CD4^+^ T cells for therapy. Interestingly, the TCR A11Vc derived from the vaccinated patient recognized a nonamer KRAS peptide, while the other three TCRs recognized a decamer KRAS peptide. This finding is important because targeted mass spectrometry revealed that the decamer was more abundantly presented than the nonamer at the surface of the cancer cell lines tested, suggesting that TCRs targeting the decamer KRAS G12V peptide may be more effective than nonamer targeting TCRs due to a higher density of target antigen.

## Considerations and outlook

Four KRAS G12V-reactive TCRs from Bear et al. (11) have the potential for use in TCR-T (Table 1). The authors selected the A11Va TCR as the lead candidate for clinical development, but additional safety testing to determine whether the TCRs recognize any other HLA molecule in an antigen-independent manner (known as HLA alloreactivity) would be prudent. Benchmarking the antitumor activity of the TCRs against other published HLA-A*03:01– and HLA-A*11:01–restricted KRAS G12V-targeting TCRs (12, 13) would be interesting. It also is unknown whether vaccination of patients is a superior approach to obtain potent antitumor mKRAS-reactive TCRs compared with current in vitro or preclinical in vivo methods. However, even when equipped with seemingly optimal TCRs, TCR-T faces challenges. Expression of the targeted antigen can be heterogeneous and low, which could lead to suboptimal tumor recognition in vivo. Indeed, the abundance of the targeted KRAS G12V peptide/HLA complex on the surface of in vitro–cultured tumor cells in this study was variable, often between four and 242 copies per cell even in cell lines that were engineered to constitutively express the restricting HLA molecule. Targeting a single antigen with TCR-T runs the risk of tumor evasion through downregulation or genetic loss of the restricting HLA element, a phenomenon observed in some patients treated with TCR-based cell therapy (8, 10, 17). Overlayed on top of these and other tumor-intrinsic factors is the suppressive tumor microenvironment (TME).

While these challenges may be daunting, there is reason for optimism. Even when targeting a single antigen, TCR-T has mediated clinical responses in some patients, perhaps best exemplified by the FDA approval in August 2024 of the first-ever TCR-T product, which targets MAGE-A4, for patients with synovial sarcoma. Thus, there is opportunity for improvement, and a multitude of innovative strategies are being investigated to enhance TCR-T efficacy. More potent T cells can be generated through modification of cell culture conditions and/or genetically knocking in or knocking out molecules that modulate T cell function, survival, or proliferation, and/or stimulate other cells within the TME. Harnessing HLA-II–restricted TCRs from CD4^+^ T cells may promote systemic antitumor immunity and overcome defects in HLA-I expression in tumors, and on its own, CD4^+^ TCR-T has mediated tumor regression in humans (18). Tumor heterogeneity could be addressed by targeting multiple patient-specific tumor antigens using a TCR-T product containing multiple TCRs (19, 20). TCR-T also could be combined with the in vivo administration of agents that may promote T cell function, such as cytokines, vaccines, immune-checkpoint inhibitors, immune agonists, and tumor-targeting small molecules, such as inhibitors or proteolysis-targeting chimeras (PROTACs). But regardless of how TCR-T is enhanced, at the core of any effective TCR-T are the requirements for a good tumor target and a good TCR. Clinical translation of Bear et al. (11) will determine whether KRAS G12V and any of the four specific TCRs fulfill these requirements and will provide additional insights into the druggability of the undruggable KRAS by T cells (9).

## Figures and Tables

**Table 1:**
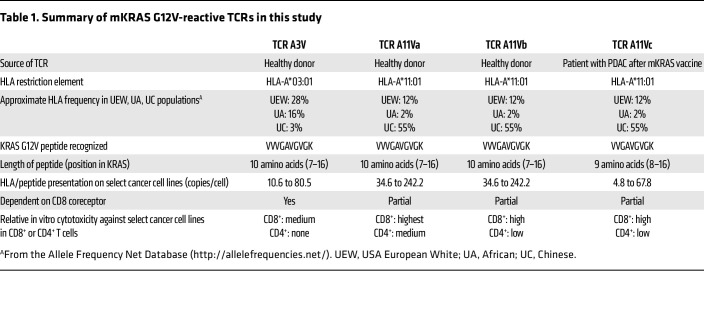
Summary of mKRAS G12V-reactive TCRs in this study

## Version 1 11/01/2024

Electronic publication
